# Temporal dynamics of plant movement under task constraints

**DOI:** 10.1186/s40462-026-00680-8

**Published:** 2026-07-10

**Authors:** Silvia Guerra, Valentina Simonetti, Emanuele Cannizzo, Umberto Castiello

**Affiliations:** Department of General Psychology (DPG), University of Padova, Padova, Italy

**Keywords:** Movement time, Kinematics, Clasping, Climbing plants, Plant movement

## Abstract

**Background:**

Movement time (MT) reflects how organisms adjust their movements to environmental constraints. In climbing plants, successful attachment to external supports depends on the temporal organization of their exploratory movements (i.e., circumnutation). Yet it remains unclear whether MT in plants varies as a function of different support properties such as its distance and width, and how multiple constraints jointly shape movement execution.

**Methods:**

To this end, pea plants were tested with supports differing in width (1.1 vs. 2 cm) and distance (5 vs. 10 cm). MT, circumnutation kinematics (i.e., duration, length and total of circumnutation) and morphological measures were analysed.

**Results:**

Pea plants modulated the temporal dynamics of their approach-to-clasp movement in response to different task constraints. Specifically, MT increased as both support distance and support width increased, indicating that these factors acted as cumulative spatial constraints.

**Conclusion:**

These findings suggest that spatial constraints modulate the temporal dynamics of pea plant approach-to-clasp movements providing a basis for future investigations into how environmental factors shape plant behaviour.

**Supplementary Information:**

The online version contains supplementary material available at https://doi.org/10.1186/s40462-026-00680-8.

## Introduction

Movement time (MT) is a fundamental feature of behaviour across biological systems because it reflects how organisms adjust their movements to environmental constraints. In animals, one of the most influential frameworks for describing the relationship between MT and task demands is the Fitts’s law [1]. This principle states that the time required to reach a target depends on both the distance to the target and its width. Specifically, MT increases with greater distance and decreases with larger target width. Mathematically, the Fitts’s law is commonly represented using the following equations:

$$\:MT=a+b\cdot\:{log}_{2}\left(\frac{2A}{\mathrm{W}}\right)$$

$$\:ID=\:{log}_{2}\left(\frac{2A}{\mathrm{W}}\right)$$

where *MT* denotes the movement time required to complete the task, *A* represents the distance to the target, and *W* corresponds to the width of the target. The constants *a* and *b* are empirically derived parameters that depend on the specific experimental conditions, or the device used. The term log_2_(2 A/W) is referred to as the Index of Difficulty (ID), capturing the task’s level of challenge by combining spatial constraints and target width.

Originally developed to account for human motor behaviour, the relationship between MT and task constraints has subsequently been investigated in a variety of animal species, including non-human primates, rats and insects [1–11]. A central question is whether this principle might be observed in other biological systems lacking a central nervous system (CNS) such as plants. Climbing plants provide a suitable model for addressing this question. Climbing plants possess weak stems and therefore depend on external supports to achieve vertical growth and maximize light exposure [12–32]. During growth, they perform circumnutation, a continuous oscillatory movement that explores the surrounding environment and increases the likelihood of encountering suitable supports [15, 16, 33]. Upon support contact, adjustments in growth direction and dynamics facilitate attachment and subsequent climbing [12–33]. Accordingly, the temporal organization of movement is biologically relevant, as successful attachment often depends on the timely coordination of exploratory and approach behaviours [12–24, 28–31]. Competition with neighbouring vegetation further increases the importance of efficient movement execution, as delays in attachment may result in the loss of valuable structural resources [12, 15]. Previous studies have shown that support characteristics, particularly thickness, influence movement dynamics and timing, with plants generally approaching thinner supports more rapidly than thicker ones [13, 19, 20, 22]. However, it remains unknown whether movement timing is similarly modulated by other spatial constraints, such as target distance, or how multiple constraints jointly shape movement execution. To our knowledge, the role of target distance in regulating movement timing has not yet been investigated in climbing plants. Determining whether distance and support thickness influence the organization of plant movement at the system level is therefore essential for understanding the principles underlying its temporal organization. To address this question, the present study investigated whether MT systematically varies as a function of support distance and width. Pea plants were tested with supports differing in width (1.1 vs. 2 cm) and distance (5 vs. 10 cm). If plant movements followed the pattern predicted by the classical formulation of Fitts’s law, MT should increase with task difficulty, resulting in longer durations for more distant and narrower supports and shorter durations for closer and wider supports. However, because clasping wider supports may entail greater growth-related and energetic costs, we anticipated that the classical formulation might not fully account for plant behaviour. Instead, we expected MT to reflect the specific biological and ecological constraints associated with establishing contact with a support.

## Materials and methods

### Plant material

Forty snow peas *(Pisum sativum* var. saccharatum cv Carouby de Maussane) were chosen as model plants. *P. sativum* seeds were selected, potted and kept at the conditions outlined below (see *Germination and growth conditions* section). Only plants that successfully approached and clasped the support were included in the analyses. This criterion was adopted because the primary aim of the study was to characterize the kinematical features of successful approach-to-clasp behaviour under different environmental conditions. Accordingly, ten individuals were analysed in each experimental condition. Consequently, the present study describes variation among successful approach-to-clasp events and does not permit conclusions regarding condition-dependent differences in the probability of approaching and clasping the support.

### Types of support

**Fig. 1:**
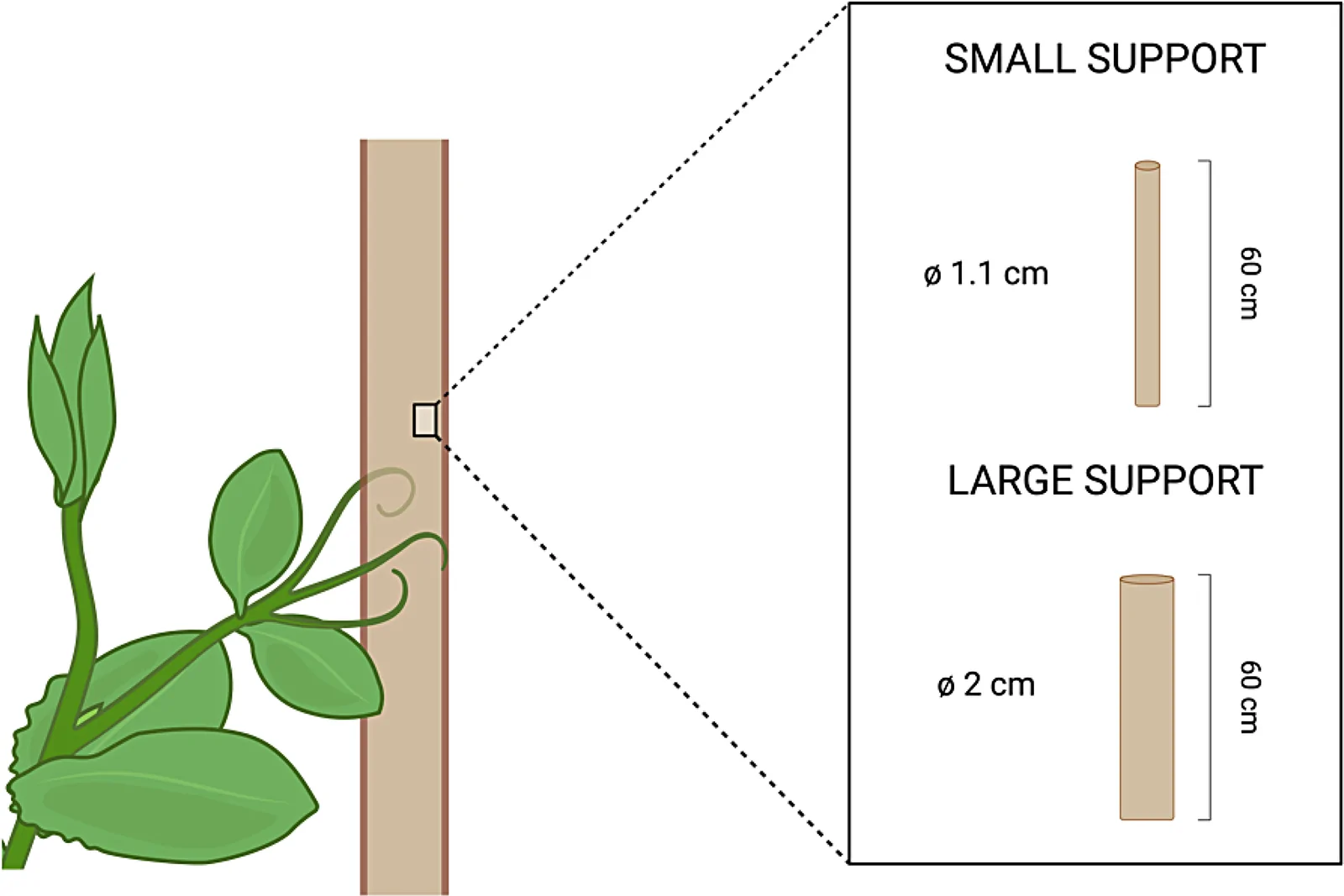
Graphical representation of the types of support used for each experimental condition

Two types of support were considered:

i). Small support (i.e., S): a 60-cm tall wooden pole with a width of 1.1 cm, inserted 7 cm below the sand soil surface (Fig. 1).
ii). Large support (i.e., L): a 60-cm tall wooden pole with a width of 2 cm, inserted 7 cm below the sand soil surface (Fig. 1).

### Experimental conditions

**Fig. 2:**
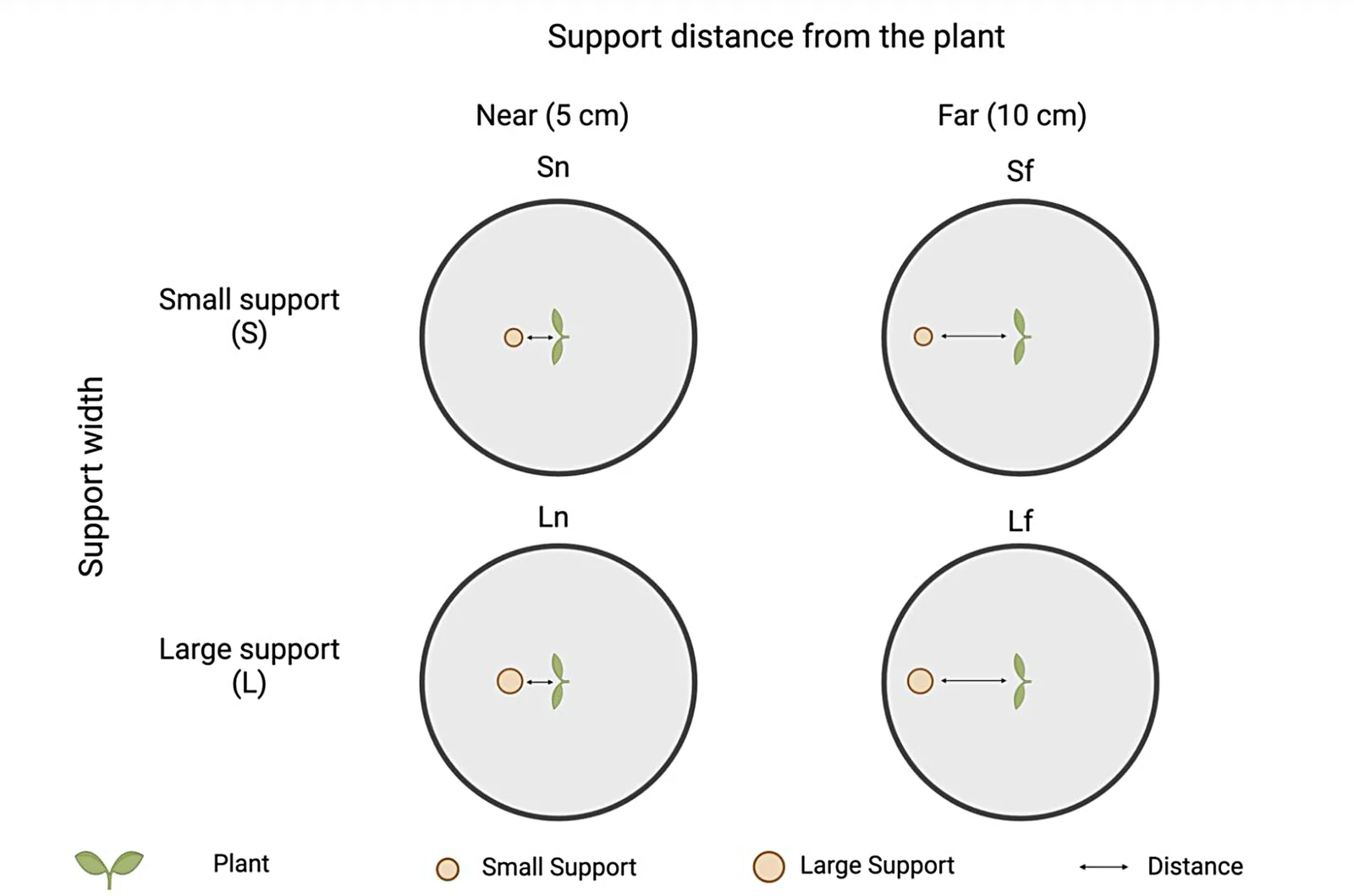
Graphical representation of the experimental conditions. Pea plants were tested in the presence of a small or large support positioned at 5–10 cm away from the plant

Plants underwent four experimental conditions:

A. *Small-near support condition* (Sn): pea plants were tested in the presence of a small support positioned 5 cm away from the plant (Fig. 2).
B. *Small-far support condition* (Sf): pea plants were tested in the presence of a small support positioned 10 cm away from the plant (Fig. 2).
C. *Large-near support condition* (Ln): pea plants were tested in the presence of a large support positioned 5 cm away from the plant (Fig. 2).
D. *Large-far support condition* (Lf): pea plants were tested in the presence of a large support positioned 10 cm away from the plant (Fig. 2).

### Germination and growth conditions

**Fig. 3:**
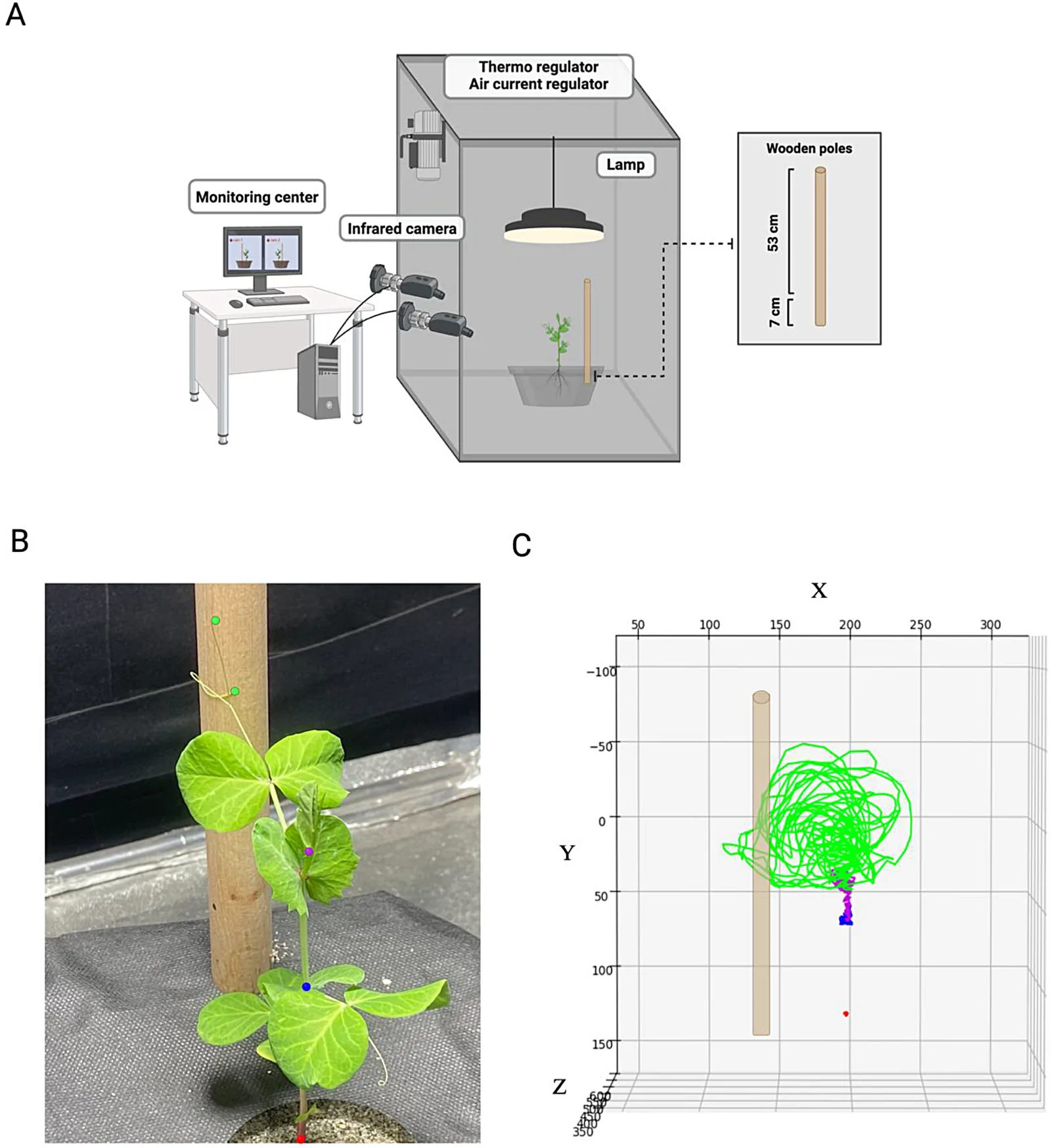
(**A**) Schematic of the experimental setup. (**B**) Anatomical landmarks tracked during the analysis: the apex (purple dot) and the tendril tips (i.e., the filamentary organs through which the plant clasps a potential support; green dots), together with the reference points used: the plant origin (red dot), the second leaf (blue dot), and the support. (**C**) Reconstructed three-dimensional trajectories of the clasping leaf’s tendril in the Ln condition, plotted along the sagittal (x), vertical (y) and medio-lateral (z) axes (mm). Across all conditions, tendril movement was consistently oriented toward the support location to approach and clasp it

Germination and growth procedures, together with the growth box configuration and environmental control, followed those detailed in our previous study [24]. Briefly, seeds were soaked in tap water for 24 h and then placed in absorbent paper for 5 days to germinate in darkness at 26 °C. Once the seeds germinated, healthy seedlings of the same root and shoot length were chosen and transplanted at the centre of the pot. Cylindrical pots (diameter 20 cm, height 20 cm) were filled with silica sand (type 16SS, size 0.8/1.2 mm, density 1.4 kg l-1). The pots were watered and fertilized using a half-strength solution culture (Murashige and Skoog Basal Salt Micronutrient Solution; 10×, liquid, plant cell culture tested; SIGMA Life Science, Milan, Italy) three times a week from plant transplanting until the end of the experimental session. Each plant was grown and tested individually in a single growth box (Cultibox SG combi, 80 × 80 × 160 cm; Fig. 3A) maintained at 26 °C. A two-fan system (extractor fan with thermo-regulator, TT125, 125 mm; input fan, Blauberg Tubo 100) ensured a steady airflow with a mean air residence time of 60 s, directed so as not to interfere with plant movement. Plants were grown under an 11 h 15 min light / 12 h 45 min dark cycle (cool-white LED illumination; V-TAC VT-911–100 W or Samsung UFO 100 W). Lamps were positioned 50 cm above each seedling providing a photosynthetic photon flux density of 350 µmol m⁻² s⁻¹ (LI-190R quantum sensor), measured at the centre of the pot across all boxes at the beginning of the study. The light-dark cycle was synchronized with the natural daily photoperiod rather than with the onset of individual experimental sessions. All plants entered the experimental procedure during the light phase, typically between late morning and early afternoon. To minimize light gradients and avoid directional phototropic cues, reflective Mylar^®^ film was applied to all box walls. As a result, the support structures did not represent localized light sources relative to the surrounding environment. Furthermore, a black non-woven fabric template was placed on the soil surface. The template contained predefined holes indicating where the seed and the support should be positioned, ensuring a consistent distance between them across all experimental trials while preserving soil aeration (Fig. 3B). Across experimental runs, treatment assignments to boxes were randomized, preventing any experimental condition from being consistently associated with a particular box.

### Video recording and data analysis

The video recording set-up, calibration and tracking procedure are reported in detail in our previous studies [24, 31] and are summarized here. Within each growth box, a pair of RGB-infrared cameras (IP 2.1 Mpx outdoor varifocal IR 1080P) was mounted 110 cm above the ground and 45 cm apart to capture stereo images of the plant. The cameras were connected via Ethernet to a 10-port wireless router (D-link Dsr-250n) and, through it, to a PC, with frame acquisition managed by CamRecorder software (Ab.Acus s.r.l., Milan, Italy). To improve the contrast between the plant’s anatomical landmarks (e.g., the apex) and the background, parts of the box walls were covered with black felt velvet and the wooden supports were darkened with charcoal. Camera calibration and depth extraction relied on a chessboard (18-mm squares, 10 × 7) imaged from multiple angles and distances, with the same chessboard placed at the centre of the box for stereo calibration. Frames were acquired synchronously every 3 min (0.0056 Hz). Plant movement was tracked without attaching any physical markers to plants. Instead, virtual markers were placed on the anatomical landmarks of interest (i.e., apex, junction of the tendrils, tendril tips) and on the reference points (i.e., lowest and highest points of the support, plant origin, second leaf, internode) using the ROOMORS-TRACKING software, a graphical evolution of the SPROUTS software (Ab.Acus s.r.l., Milan, Italy; Fig. 3B) [31]. Tracking was first performed automatically over the whole movement sequence with the Kanade-Lucas-Tomasi algorithm, after distortion removal, and then verified frame-by-frame by the operators. Each marker’s 3D trajectory was reconstructed by triangulating the 2D trajectories from the two cameras (Fig. 3C) [31]. For each experimental condition, the leaf that touched the support was considered in the analysis. The initial movement was defined as the frame in which the tendrils (i.e., filamentary organs used by plants to clasp a potential support) started to develop and were clearly visible from the apex. The end of the movement was defined as the frame in which the tendril(s) contacted the support. The end point was visually inspected frame-by-frame to identify the exact moment at which the tendrils first contacted the support.

### Kinematical dependent variables

The dependent variable specifically tailored to test our hypothesis, based on our previous studies [20–24], was the MT (min; the interval between the onset of tendril movement and the first contact of the tendril with the support). This measure was chosen because it quantifies the total time the plant spends executing the approaching and clasping behaviour towards the support. The present study considered only the approaching and touching phase toward the support, whereas the subsequent coiling and anchorage phases were not examined.

Furthermore, the movement of pea plants is characterized by circumnutation, a growth pattern consisting of an oscillatory rotation of the organs around their central axis [15, 16]. Climbing plants rely on this movement to explore the surrounding and to locate elements essential for their survival, such as potential supports [32, 33]. Circumnutation is a physiologically relevant mechanism through which plants adjust their growth to environmental conditions by varying the number, length, velocity and duration of their oscillations [21, 22]. Within this framework, circumnutation parameters may help to interpret the mechanisms underlying the modulation of MT as a function of task difficulty. Specifically, under conditions of greater task difficulty, an increase in MT may reflect the execution of a larger number of circumnutations, of greater amplitude and longer duration. Such dynamic modulation of circumnutation may enable plants to explore the environment more extensively. Accordingly, the following dependent variables were also considered:

i. The total number of circumnutations: the overall count of the circumnutations performed by the plant during the entire MT.
ii. The length of the circumnutation (mm): the sum of the Euclidean distances between consecutive points along the trajectory of a circumnutation.
iii. The duration of the circumnutation (min): the time taken by the tendrils to complete a single circumnutation.

### Morphological measurements

**Fig. 4:**
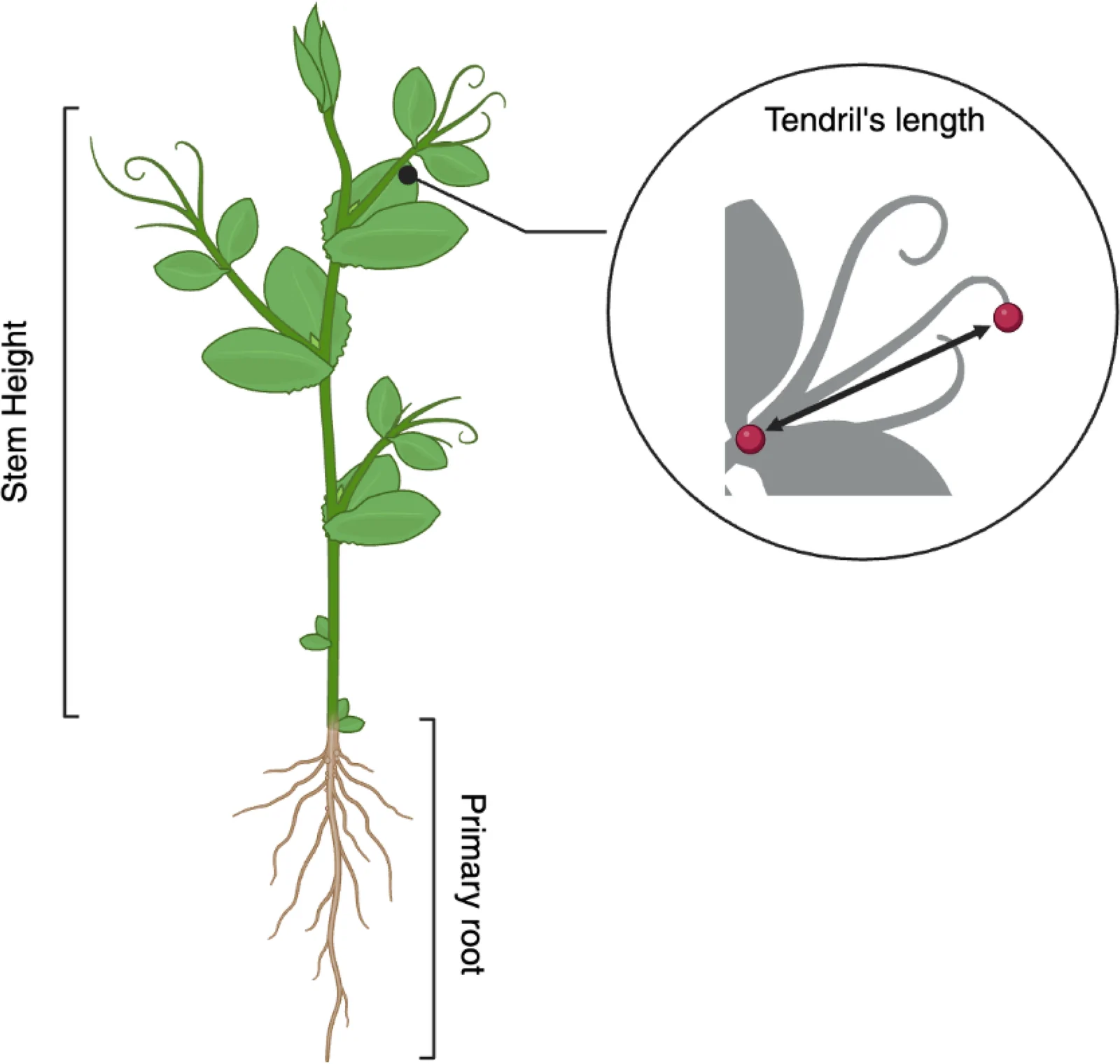
Graphical representation of the morphological measurements

Aboveground measurements were performed using a precision caliper with a resolution of 0.01 cm, while belowground measurement (i.e., the length of the primary root) was performed using a ruler with a resolution of 0.1 cm once the plant clasped the support (Fig. 4). Measurements included: (i) stem height (cm), (ii) number of tendrils, (iii) length of each tendril (mm), and (iv) the primary root length (cm). Stem height was calculated as the sum of all internode lengths from the origin of the stem to the apical meristem (Fig. 4).

Tendril length was measured as the distance from the node of origin of the tendrils to their tips (Fig. 4). The primary root length was measured as the distance between the base of the hypocotyl to the tip of the root (Fig. 4). All morphological measurements, including stem height, were obtained at the end of the experimental session, corresponding to the moment at which the plant first clasped the support. Consequently, the duration of the observation period varied across individuals (Table S1). To further characterize plant growth during the observation period (i.e., plant age), we also report an average stem elongation rate (cm/day), calculated as final stem height divided by the duration of the experimental session (Table S1). Because initial stem length was not measured, this parameter should be interpreted as an index of average elongation over the observation period rather than a true growth rate. Nevertheless, all seedlings were selected at the onset of the experiment to be similar in size and developmental stage, thereby minimizing potential variability in initial stem length among individuals. Consequently, any bias arising from differences in starting stem length is likely to have been limited. Plant age and average stem elongation rate are reported in Table S1 for descriptive purposes only and were not included in the statistical analyses.

### Data analysis

All analyses were conducted in R environment [34] using linear mixed-effects models (LMMs) [35]. The Index of Difficulty (ID) was computed according to the classical formulation of Fitts’s law (ID = log₂(2 A/W)). Box was included as a random intercept to account for shared environmental influences among plants. The contribution of box-level variation was quantified using the variance of the random effect and the intraclass correlation coefficient (ICC). Because MT was available as a single observation per plant, plant identity was not included as a random effect. Model fit was evaluated using marginal and conditional R² values. Fixed effects were assessed using Type III F-tests with Satterthwaite’s approximation of degrees of freedom (*lmerTest package*). When significant main effects were detected, post hoc pairwise comparisons were performed using estimated marginal means with Tukey adjustment [36]. The number of observations was 40 for each model, except for the model including the primary root length, for which the sample size was 38 due to two primary roots being damaged during plant removal. The significance level was set at *p*<.05.

### Testing MT and spatial constraints in plants: A Fitts’s law inspired perspective

To test whether plant MT is influenced by task constraints, a linear mixed-effects model (lme4 package; LMM) [35] was fitted with MT as the dependent variable, ID as a fixed effect. To assess deviations from the classical formulation of Fitts’s law, additional LMM [35] including the distance from the support (*A*) and target width (*W*) as separate predictors and their interaction was fitted. Models were compared using Akaike’s Information Criterion (AIC) [37].

### Testing whether MT modulation in pea plants relates to a biological cost of growth or an exploratory control strategy

#### Growth-related morphological constraints

To examine whether MT reflected growth-related constraints, we first assessed the correlation between plant height and the primary root length using Pearson’s correlation coefficient. Linear mixed effect models (LMMs; *lme4 package*) [35] were then fitted with MT as the dependent variable and ID, height, and primary root length as fixed effects. To further examine whether morphological traits in plants differed across conditions (Lf, Ln, Sf, Sn), additional LMMs (lme4 package) [35] were conducted with height and the length of the primary root as dependent variables, and Condition as fixed effect. To assess whether morphological traits accounted for MT differences across conditions, an additional LMM [35] was fitted including Condition, height, and the length of the primary root as fixed effects.

#### Tendrils morphology as predictors of MT

The contribution of tendril morphology was assessed by including the total number of tendrils and mean tendril length as predictors of MT. Because MT was available as a single observation per plant, tendril-level measures were aggregated at the plant level. For each plant, total tendril number and mean tendril length were computed. Aggregation was performed by grouping observations by plant identity and computing summary statistics across tendrils, thereby generating a plant-level dataset suitable for mixed-effects modeling. An initial linear mixed-effects model (LMM) [35] was fitted with MT as the dependent variable and ID, the total tendril number, and mean tendril length as fixed effects. To test whether tendril morphology differed across experimental conditions (Ln, Lf, Sn, Sf), separate LMMs [35] were conducted with total tendril number and mean tendril length as dependent variables and Condition as fixed effect. Finally, to determine whether tendril morphology mediated condition-related differences in MT, an additional LMM [35] was fitted including Condition, total tendril number, and mean tendril length as fixed effects predicting MT.

#### Circumnutation dynamics as predictors of MT

The contribution of the circumnutation dynamics on MT was assessed by including the number of circumnutations, their length and duration as predictors of MT. Circumnutation measures were aggregated to the plant ID to allow integration with MT, which was available as a single observation per plant. For each plant, the mean measures of circumnutation parameters (number, length and duration of circumnutation) were computed. Aggregation was performed by grouping observations by plant identity and computing summary statistics across tendrils, thereby generating a plant-level dataset suitable for mixed-effects modeling. An initial LMM [35] was fitted with MT as the dependent variable and ID, total number of circumnutations, mean duration, and mean length as fixed effects. To examine whether circumnutation dynamics differed across experimental conditions (Ln, Lf, Sn, Sf), separate LMMs [35] were conducted with circumnutation number, duration, and length as dependent variables and Condition as fixed effect. Finally, to determine whether circumnutation dynamics accounted for condition-related differences in MT, an additional LMM [35] was fitted including Condition and all circumnutation parameters as fixed effects predicting MT.

#### Modeling the effect of spatial constraints in pea plant movements

A pea plant-specific Index of Difficulty was advanced to express difficulty in relative terms.

$$\:I{D}_{Plant}=\:{log}_{2}\left(\frac{A\cdot\:W}{k}+1\right)$$

where *A* represents the distance to the target, *W* corresponds to the width of the target, and *k* denotes the product between the averages of target distance and width across experimental conditions. The *k* avoids differences in scale between spatial parameters. Adding + 1 ensures that the content of the logarithm never becomes negative and biologically impossible. To test whether the ID_plant_ accounted for variation in MT, a linear mixed-effects model (LMM) [35] was fitted with MT as the dependent variable, ID_plant_ as a fixed effect. The performance of the pea plant-specific model was then compared with that of the classical Fitts model using the Akaike Information Criterion (AIC) [37], with lower AIC values indicating better model fit.

## Results

### Qualitative results

The apex and the tendrils showed a growing movement pattern characterized by circumnutation (Fig. 3C). In all conditions, as the plant grew, it started to move towards the support and the tendrils began to assume a choreography suited to clasping it (Video S1-S4).

### Testing MT and spatial constraints in plants: A Fitts’s law inspired perspective

**Fig. 5:**
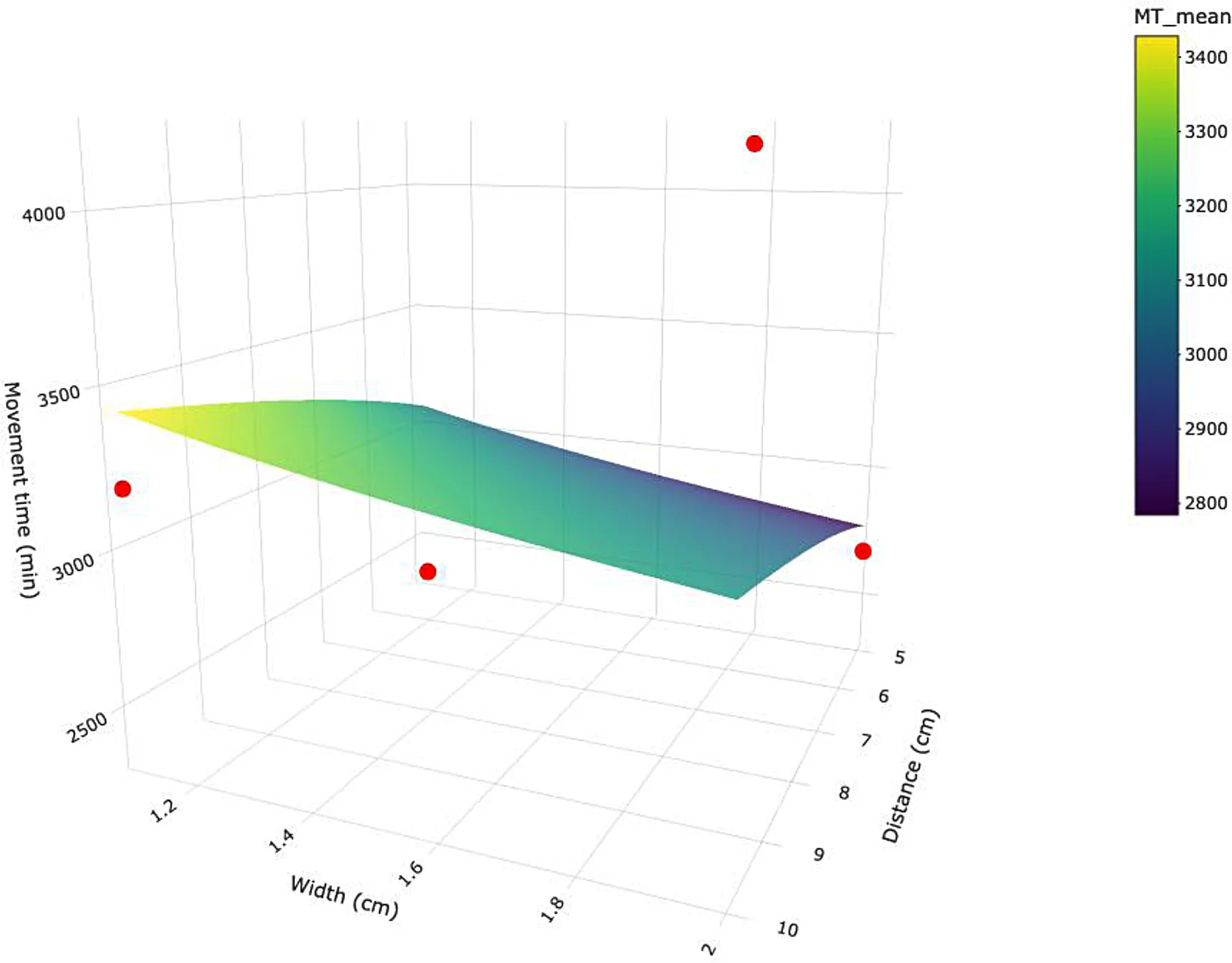
Three-dimensional regression surface of MT as a function of target distance (*A*) and target width (*W*). The surface represents the predicted values from the linear model based on the Index of Difficulty (ID), computed according to Fitts’s law formulation [ID = log₂(2 A/W)]. Red markers indicate the observed mean MT for each experimental condition. The surface reflects the linear trend predicted by Fitts’s law. Observed condition means deviate from the fitted plane, suggesting that the classical formulation does not fully account for the variability in MT across conditions

Results showed a positive but non-significant relationship between MT and the classical formulation of the ID (β = 358.59, t(38.22) = 1.45, *p* = .154; R^2^ₘ=0.05; R^2^_c_=0.08). The variance associated with the Box random intercept was 28,774 min² (SD = 169.6 min), corresponding to an ICC of 0.027, indicating that only 2.7% of the total variance in MT was attributable to differences among growth boxes. MT tended to increase with the ID, consistent with the predictions of Fitts’s law, although the effect did not reach statistical significance (Fig. 5; Table S2).

### Decomposing the ID: Independent contributions of distance (A) and width (W)

We further decomposed the ID into distance (*A*) and target width (*W*) to examine whether these factors independently and/or additively influenced MT, as predicted by classical Fitts’s law. Within the range of support width and distance tested in the present experiment, both *A* and *W* significantly modulated MT. Specifically, MT increased with both distance (log₂A: β = 1179.0, t(40) = 4.55, *p* < .001; Table S3) and width of the target (log₂W: β = 774.6, t(40) = 2.58, *p* = .014; Table S3). Notably, the positive effect of *W* is opposite to the classical prediction of Fitts’s law, in which larger targets are expected to reduce MT. To test whether the effects of *A* and *W* deviated from the additive structure predicted by Fitts’s law, an interaction between log₂(A) and log₂(W) was included in the model. The interaction was not significant (β = 761.7, t(40) = 1.30, *p* = .202; Table S3), suggesting that the effects of *A* and *W* combined additively. The variance associated with the Box random intercept was estimated as zero in both models, indicating no detectable box-level variation in MT after accounting for the fixed effects included in the model. A comparison between the additive and interaction models showed no significant improvement when including the interaction term (χ²(1) = 1.64, *p* = .200). Model comparison based on Akaike’s Information Criterion (AIC) [37] indicated that models including *A* and *W* as separate predictors, either additively (AIC = 660.1) or with their interaction (AIC = 660.5; Table S4) provided a more parsimonious statistical fit than the Fitts model (AIC = 677.03). However, despite its better global fit, the direction of *W* effect revealed a deviation from the classical Fitts’s law formulation. Together, these findings suggest that plant movement does not conform to the classical relationship between MT and task difficulty and instead may reflect either increased energetic costs associated with growth and/or an adaptive exploratory strategy.

### Testing whether MT modulation in pea plants relates to a biological cost of growth or an exploratory control strategy

#### Growth-related morphological constraints

**Fig. 6:**
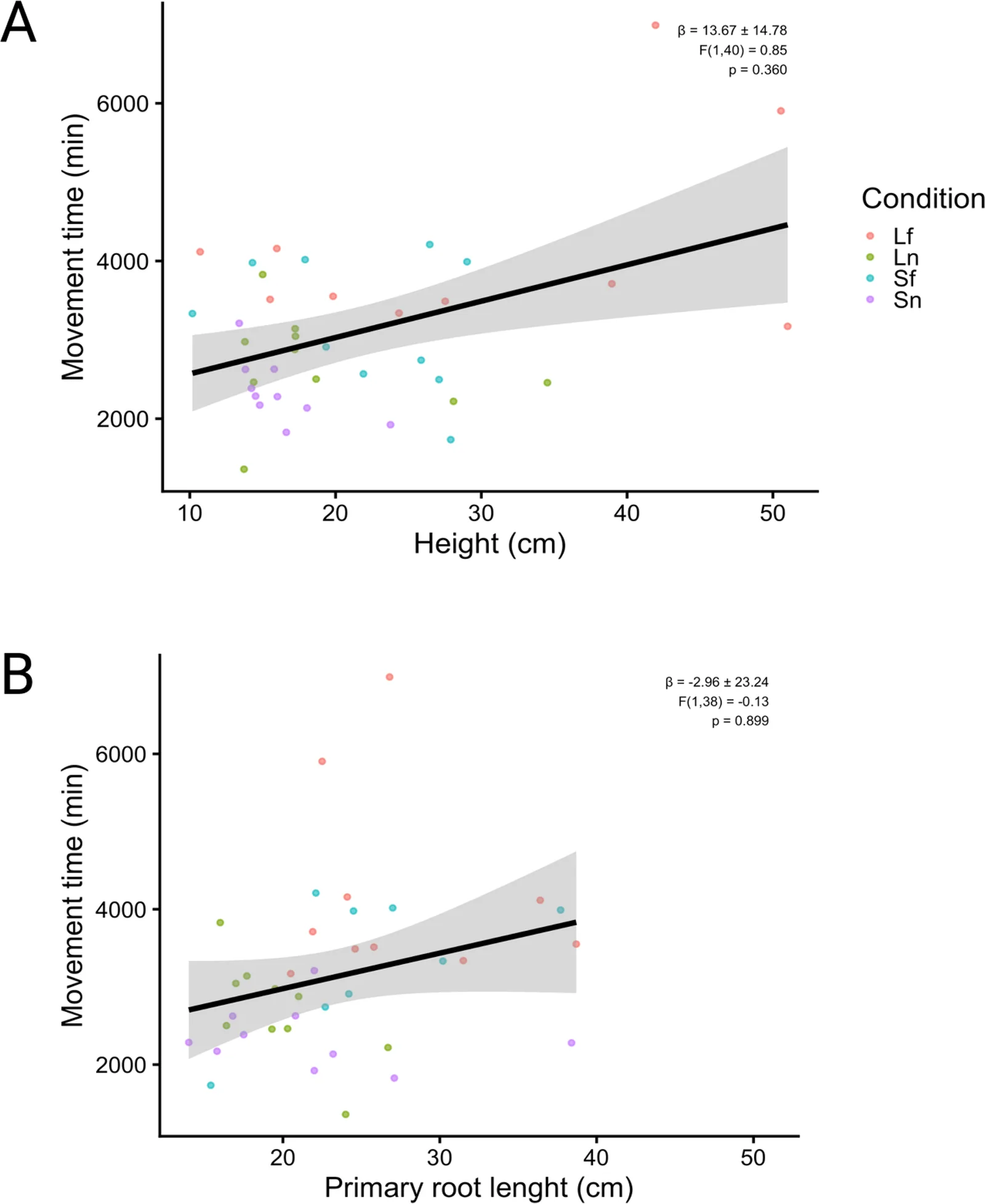
Relationship between MT - height **(A)** and MT -primary root **(B).** Points are coloured by experimental condition; the solid line represents the overall regression (95% CI)

To investigate whether the modulation of MT reflected a biological cost of growth and/or an adaptive control strategy, we first examined the contribution of growth-related factors on MT (Table S1). No significant correlation was found between the height and the primary root length (*r* = .002, *p* = .992), suggesting that the two morphological traits varied independently. In the full model including the classical ID, stem height, and the length of the primary root (Table S5), stem height significantly predicted MT (F(1,36.27) = 9.25, *p* = .004). In contrast, the primary root length (F(1,33.40) = 2.40, *p* = .131) and ID (F(1,34.54) = 1.01, *p* = .321) were not significant predictors (R²ₘ = 0.29; R²c = 0.31). The variance associated with the Box random intercept was 23,159 min² (SD = 152.2 min), corresponding to an adjusted ICC of 0.028, indicating that approximately 2.8% of the total variance in MT was attributable to differences among growth boxes. We then tested whether experimental condition influenced plant morphology. Stem height significantly differed across conditions (F(3,40) = 4.71, *p* = .007, R²ₘ = 0.27, R²c=0.27; Table S6) with plants in the Lf condition showing greater height compared to the Sn condition (Table S7). The length of the primary root also varied across conditions (F(3,38) = 3.61, *p* = .022, R²ₘ = 0.23, R²c=0.23; Table S6). However, post hoc pairwise comparisons did not reveal significant differences between specific conditions (Table S7). To determine whether morphology accounted for differences in MT across conditions, we fitted models including both condition and morphological variables. When stem height, the length of the primary root and experimental condition were considered (Table S8), experimental condition remained a significant predictor of MT (F(3,38) = 4.57, *p* = .008), whereas height (F(3,38) = 1.07, *p* = .307) and the length of the primary root (F(3,38)= 0.02, *p* = .882) were no longer significant (R²ₘ = 0.47, R²c=0.47; Table S8). Post hoc pairwise comparisons revealed that Lf condition exhibited significantly longer MT than Ln (Table S9). The variance associated with the Box random intercept was estimated as zero in all three models, indicating no detectable box-level variation in MT after accounting for the fixed effects included in the model. Overall, despite condition-dependent differences in morphology, the structural traits considered in the present study did not account for variation in MT. MT remained associated with experimental condition after controlling for these morphological variables, suggesting that factors beyond the measured growth-related traits contribute to its modulation (Fig. 6A, B). However, the present analyses do not allow a definitive separation of growth-related constraints from adaptive movement processes, preventing a clear assessment of their respective contributions to variation in MT.

#### Tendrils morphology as predictors of MT

We investigated whether and to what extent tendril morphology contributed to the modulation of MT. Pea plants developed a variable number of tendrils with different lengths across individuals (Table S1), potentially reflecting differences in how plants explore and interact with the environment. We therefore tested whether variation in tendril number and length was associated with changes in MT, as a possible morphological component of exploratory control strategies adopted under different task constraints. When ID, mean tendril length, and number of tendrils were included as predictors, none of these variables significantly explained MT (ID: F(1, 38.19) = 1.94, *p* = .172; mean tendrils length: F(1, 39.88) = 0.08, *p* = .784; number of tendrils: F(1, 39.87) = 0.03, *p* = .862, R²ₘ = 0.05; R²c = 0.07; Table S10). The variance associated with the Box random intercept was 20,072 min² (SD = 141.7 min), corresponding to an ICC of 0.019, indicating that only 1.9% of the total variance in MT was attributable to differences among growth boxes. We then tested whether experimental condition influenced tendrils morphology. Both tendril length (F(3, 40) = 7.34, *p* < .001, R²ₘ = 0.36, R²c=0.36) and tendril number (F(3, 40) = 3.95, *p* = .015, R²ₘ = 0.23, R²c=0.23) varied significantly across conditions (Table S11), with longer tendrils in Ln and Sn compared to Sf, and a greater number of tendrils in Sf relative to Sn (Table S12). However, when experimental condition was included in the model, a significant main effect of Condition emerged (F(3, 40) = 14.40, *p* < .001), whereas neither mean tendril length (F(1, 40) = 2.46, *p* = .124) nor number of tendrils (F(1, 40) = 4.06, *p* = .051) significantly predicted MT (Table S13). Post hoc comparisons confirmed longer MT in Lf compared to Ln and Sn, and in Sf compared to Sn (Table S14). The variance associated with the Box random intercept was estimated as zero in the above three models, indicating no detectable box-level variation in MT after accounting for the fixed effects included in the model. These findings indicate that although tendril morphology varies across experimental conditions, it does not directly account for variation in MT once task constraints are considered. This pattern is consistent with the possibility that MT may be influenced by dynamic exploratory processes in addition to structural constraints, although the present analyses do not allow these contributions to be fully disentangled.

#### Circumnutation dynamics as predictors of MT

To further investigate factors associated with variation in MT, we examined whether circumnutation dynamics accounted for variation in MT (Table S15). In a model including circumnutation parameters and the classical ID, only the total number of circumnutations significantly predicted MT (F(1, 36.82) = 21.43, *p* < .001; Table S16). Neither circumnutation length (F(1, 39.62) = 0.13, *p* = .723; Table S16) nor mean circumnutation duration (F(1, 35.78) = 0.02, *p* = .899; Table S16) were significant predictors of MT. The classical Index of Difficulty was also not significant in this model (F(1, 34.45) = 0.95, *p* = .337, R²ₘ = 0.38, R²c = 0.46; Table S16). The variance associated with the Box random intercept was 86,190 min² (SD = 293.6 min), corresponding to an ICC of 0.120, indicating that approximately 12.0% of the total variance was attributable to differences among growth boxes. Furthermore, we examined whether exploratory strategies of plants differed across experimental conditions. Circumnutation duration differed significantly across conditions (F(3, 36.88) = 13.56, *p* < .001, R²ₘ = 0.49, R²c = 0.56; Table S17), with longer circumnutation in the Lf condition compared to Ln and Sn (Table S18), and in Sf compared to Sn (Table S18). The total number of circumnutations also varied across conditions (F(3, 40) = 4.20, *p* = .011, R²ₘ = 0.24, R²c = 0.24; Table S17), with fewer circumnutations in the Sn condition compared to the Lf condition (Table S18). No significant differences were observed in the length of the circumnutation across conditions (F(3, 36.80) = 1.02, *p* = .396; R²ₘ = 0.06, R²c = 0.20; Table S17). For circumnutation duration, the variance associated with the Box random intercept was 37.33 min² (SD = 6.11 min), corresponding to an adjusted ICC of 0.132, indicating that approximately 13.2% of the total variance was attributable to differences among growth boxes. Similarly, for circumnutation length, the variance associated with the Box random intercept was 19.95 min² (SD = 4.47 min), corresponding to an adjusted ICC of 0.142, indicating that approximately 14.2% of the total variance was attributable to differences among growth boxes. In contrast, for the total number of circumnutations, the variance associated with the Box random intercept was estimated as zero, indicating no detectable variation among growth boxes after accounting for the fixed effects included in the model. Crucially, when circumnutation dynamics were included in models predicting MT across conditions (Table S19), both circumnutation duration (F(1, 39.68) = 10.53, *p* = .002), and the number of circumnutations (F(1, 35.15) = 7.77, *p* = .008) significantly predicted MT with longer and more frequent circumnutation associated with shorter MT (Table S19). The circumnutation length did not significantly predict MT (F(1, 39.87) = 0.25, *p* = .619; Table S19). Post hoc comparisons showed an increase in the MT for the Lf condition compared to the Ln, Sf and Sn (Table S20). Furthermore, longer MT was observed in Sf condition compared to Sn and Ln conditions (Table S20). For this model, the variance associated with the Box random intercept was 57,044 min² (SD = 238.8 min), corresponding to an adjusted ICC of 0.137, indicating that approximately 13.7% of the total variance was attributable to differences among growth boxes.

#### Modeling the effect of spatial constraints in pea plant movements

**Fig. 7:**
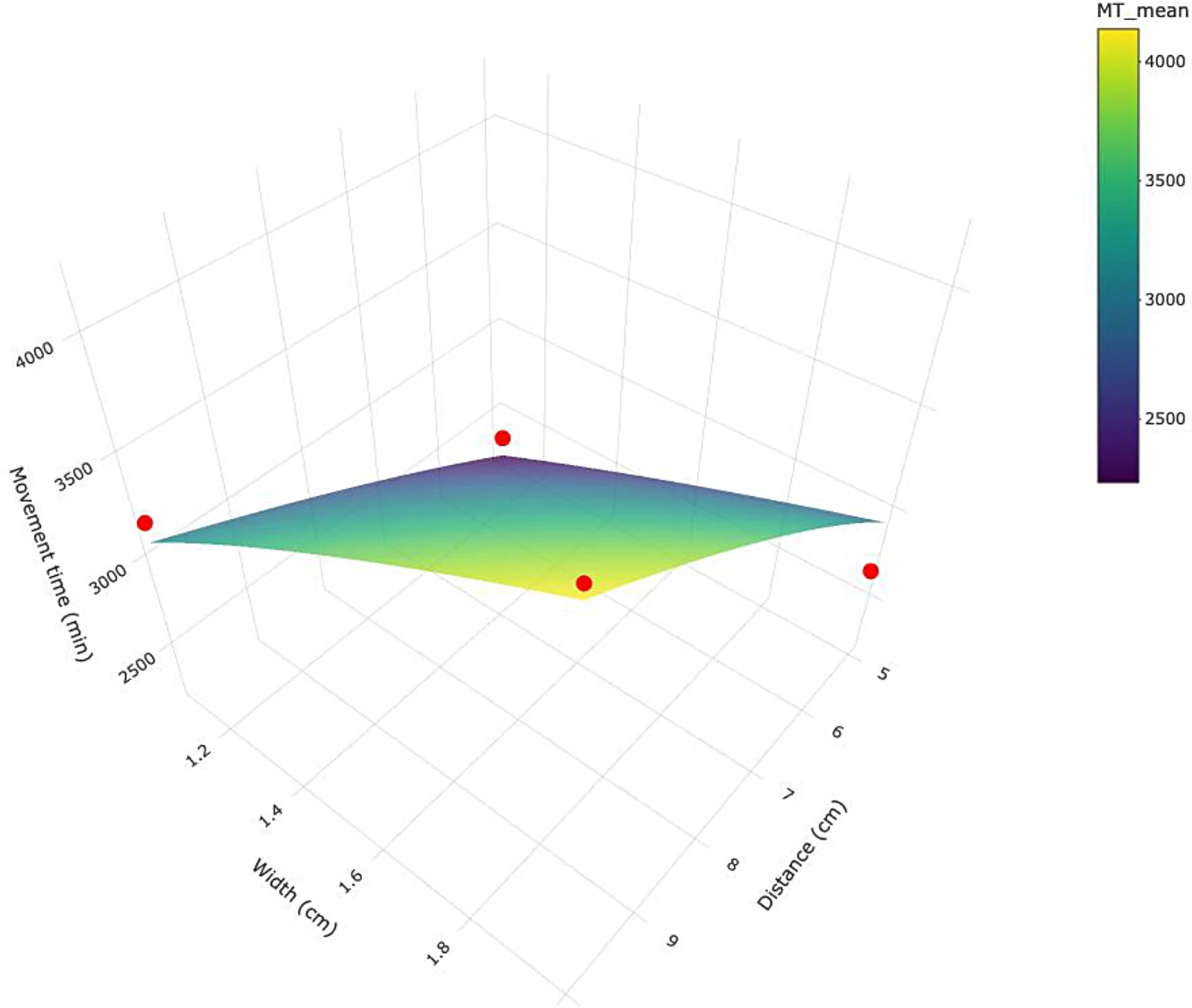
Three-dimensional regression surface of MT as a function of target distance (A) and target width (W). The surface represents the values predicted by the linear model based on the pea plant-specific Index of Difficulty (IDplant = log₂(1 + (A·W)/k)). Red markers indicate the observed mean MT for each experimental condition. The surface approximates the overall relationship between MT and the spatial constraints imposed by target distance and width, although there are some condition-specific deviations. MT in the LN condition is moderately overestimated, but it is important to note that the points shown in the graph represent the observed sample mean for that condition and not a theoretical population value, thus some deviation between the fitted surface and individual condition means is expected

Overall, the results suggest that variation in MT could not be readily explained by the growth-related measures considered in the present study. At the same time, plants exhibited systematic changes in the temporal dynamics of their movements, engaging more prolonged (i.e., circumnutation duration) and repeated exploratory (i.e., total number of circumnutations) behaviors as task difficulty increased. Furthermore, in pea plants both target distance and support width contributed in the same direction to MT, acting as cumulative spatial constraints rather than opposing factors as assumed in the classical formulation. This pattern raises the possibility that task difficulty in pea plants may depend on the combined influence of distance and width. Together, these findings suggest that the classical formulation of Fitts’s law does not adequately describe the present data. In light of these observations, we explored whether a composite index combining these two spatial constraints could provide a parsimonious description of the observed relationship. To this end, we derived an exploratory pea plant-specific Index of Difficulty based on the product of target distance and width (*A*·*W*). To ensure that the ID was dimensionless, we normalized both *A* and *W* by their respective mean values observed in the experiment. By dividing *A* and *W* by their average values, we express each condition relative to what is typical in our experimental setup. In other words, we measured how much bigger or smaller the effect of task difficulty is compared to the average task, instead of considering its absolute size into a specific unit (cm in our study). This allows the index to reflect relative differences in task demands. As a result, the index can be compared across experiments, even if the absolute distances and widths are different. Following above reasonings, we defined a pea plant-specific Index of Difficulty as:

$$\:I{D}_{Plant}=\:{log}_{2}\left(\frac{A\cdot\:W}{k}+1\right)$$

where *A* reflects the distance, *W* the width of the target and *k* denotes the product between the averages of target distance and width across experimental conditions. The *k* avoids differences in scale between spatial parameters. Adding + 1 ensures that the content of the logarithm never becomes negative [38]. We then tested whether MT increased linearly as a function of this ID_plant_. Results showed that the classical Fitts’s formulation provided a poor account of plant MT, showing a positive but non-significant relationship between MT and ID (β = 358.6, t(40) = 1.45, *p* = .154; AIC = 677.0; Table S21). In contrast, the pea plant-specific Index of Difficulty was significantly associated with MT (β = 2150.1, t(40) = 5.26, *p* < .001) and substantially improved model fit (AIC = 657.0; Table S21; Fig. 7). For the classical Fitts’s formulation, the variance associated with the Box random intercept was 28,774 min² (SD = 169.6 min), corresponding to an ICC of 0.027, indicating that only 2.7% of the total variance in MT was attributable to differences among growth boxes. In contrast, for the pea plant-specific Index of Difficulty, the variance associated with the Box random intercept was estimated as zero, indicating no detectable variation among growth boxes after accounting for the fixed effects included in the model.

Model comparison revealed a ΔAIC of approximately 19.05 in favor of the pea plant-specific formulation (Table S22), suggesting that this exploratory index may provide a more parsimonious descriptive account of MT than the classical Fitts’s formulation under the present experimental conditions. Importantly, the proposed pea plant-specific index should not be interpreted as a reformulation or extension of Fitts’s law. Rather, it represents an exploratory and descriptive metric that summarizes the joint contribution of support distance and width to MT under the present experimental conditions. Additional studies including a wider range of task constraints and measures of movement accuracy will be required to determine whether more general principles underlie this relationship.

## Discussion

The present study provided a pea plant-specific analysis of how MT varies with task constraints, inspired by the classical framework of the Fitts’s law [1, 4, 6, 38–40]. Our findings show that pea plants modulate MT as a function of task demands. However, the functional relationship between spatial parameters and MT differs from the classical formulation observed in animals. MT increased as supports became more distant and wider. While the effect of distance aligns with classical predictions, the effect of width does not: larger supports led to longer, not shorter, MT. Thus, distance and width acted as cumulative spatial constraints rather than as opposing factors. These findings indicate that the canonical structure of Fitts’s law does not directly apply to plant movement. Two alternative explanations were considered. The first concerned a potential biological cost of growth: farther and wider supports might require greater biomass production and energy expenditure. Although morphological differences emerged across conditions, neither stem height nor primary root length explained variation in MT. When experimental condition was entered into the models, these morphological variables ceased to predict MT, suggesting that the observed variation in MT is not fully explained by the morphological measures that have been considered. The second explanation concerned adaptive control. Analyses of circumnutation dynamics showed that longer MT were associated with changes in exploratory behaviour, particularly in the number and duration of circumnutations. This interpretation aligns with previous work suggesting flexible and online control in plant movements [13, 14, 22]. These patterns further support the possibility that plants adjust aspects of their exploratory activity in response to increasing and combined spatial constraints. However, because growth-related processes and movement dynamics are intrinsically linked in plants, the present analyses do not allow a complete separation of growth-related constraints from potential adaptive modulation of movement.

Based on these results, we explored whether a composite measure combining distance and width could provide a parsimonious description of the observed relationship. Unlike the classical log₂(2 A/W) formulation, this exploratory measure treated distance and width as cumulative constraints and normalized them relative to their experimental means. Although this approach provided a better fit to the present data than the classical model, it should be interpreted primarily as a descriptive summary of the relationship observed under the present experimental conditions. Alternative formulations, including models treating distance and support width as separate predictors, also captured a substantial proportion of the observed variation. Future work will be needed to assess its applicability across species, tasks, and environmental conditions and to determine whether biologically informed formulations provide additional explanatory power. For instance, future refinements may incorporate differential weighting of the *A* and *W* parameters or biologically grounded constraints such as maximum reachable distance. The impact of width and distance on MT may also vary depending on the specific morphological characteristics of the species in question. Therefore, the proposed index should be regarded as a descriptive summary of the present findings rather than as a plant equivalent of Fitts’s law or a general law governing plant movement.

**Fig. 8:**
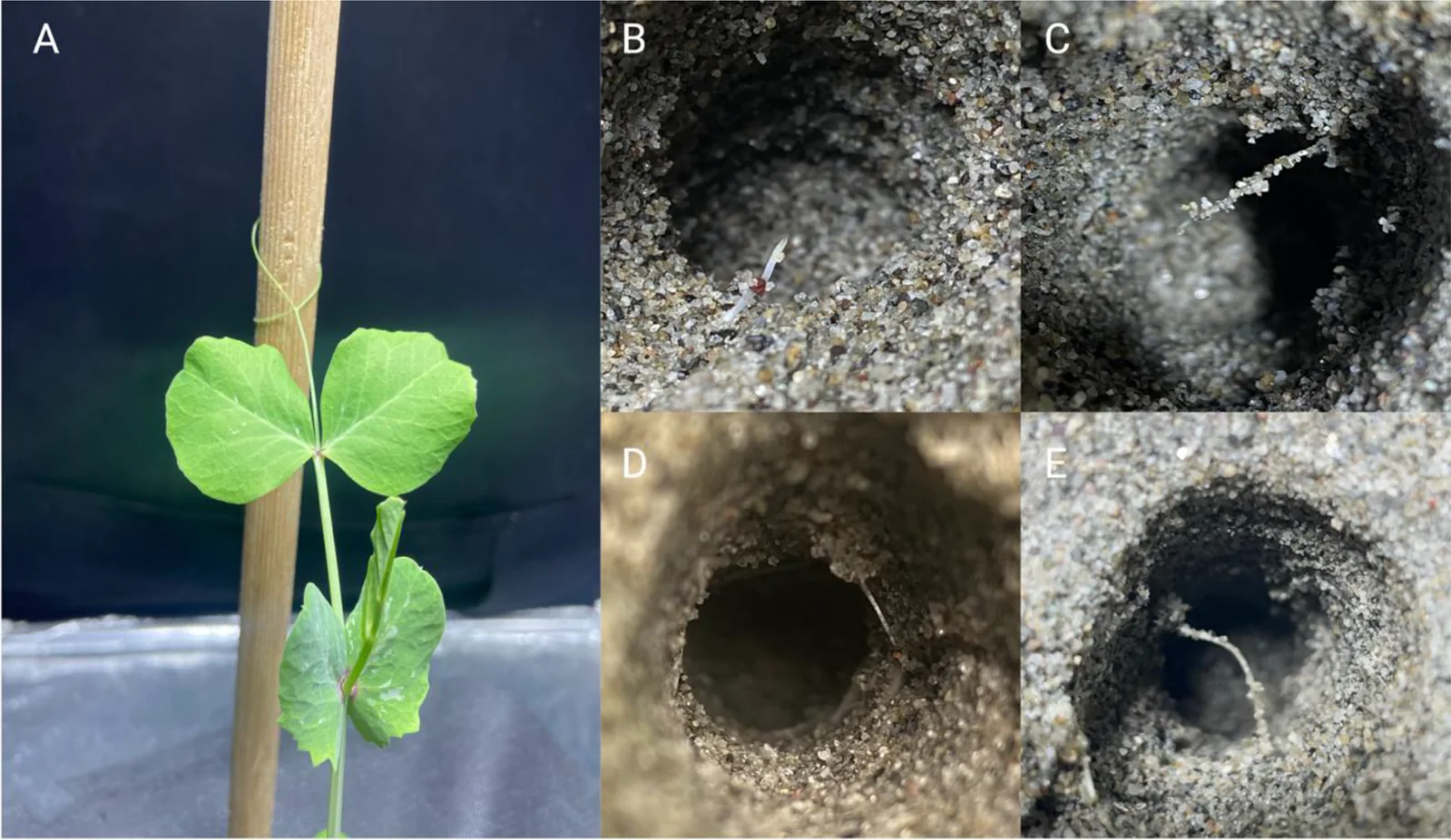
**A**) Photographs of the pea plant clasping the support for the Sn condition and of the roots clinging to the belowground part of the support for the *Sn* condition (**B**), *Ln* (**C**), *Lf* (**D**) and for the *Sf* (**E**) conditions

The present findings also raise questions about the mechanisms through which support features influence plant movement. In particular, the increase in MT as a function of support width and distance suggests that climbing plants respond not only to the presence of a support but also to its characteristics that are relevant for a successful attachment. How this is achieved without a central nervous system (CNS) remains unclear. Evidence suggests that the root system may contribute to modulating the plant’s response to the support and its properties. Several physiological mechanisms could support this process. Roots are, indeed, capable of mechanical sensing and chemical signalling through root exudates [41–45], and mechanosensitive calcium channels mediate rapid signal transduction in response to contact [46–48]. Mechanical stimulation activates mechanosensitive calcium channels, generating intracellular calcium signals that can propagate through plant tissues and convey signals in response to external stimuli [46–50]. In parallel, long-distance communication between roots and shoots is mediated by electrical activity [51–54] and phytohormonal transport, including auxin, cytokinin, strigolactones, gibberellins, and abscisic acid [55, 56], conveyed through vascular tissues [57, 58]. Together, these signalling pathways provide a plausible substrate through which signals originating belowground could influence shoot movements and attachment behaviour. Behavioural findings are consistent with this possibility. When the belowground portion of a support is inaccessible to the roots, plants are unable to localize the support and fail to appropriately adjust the kinematics of their approach movements [20, 24]. Moreover, MT is longer when access to the support is restricted to the aerial portion than when the support is available to both the aboveground and belowground organs of the plant [20, 24]. Consistent with these findings, previous experiments in which supports of different thicknesses were suspended above the soil showed that thickness-dependent kinematic modulations disappeared when the support was inaccessible to the root system, indicating that the root system may play a role in the emergence of these adjustments. At the same time, pea plants can successfully guide their approach-to-clasp behaviour by combining shoot-derived inputs from a lifted support with root-derived inputs from a grounded support, even when the two support portions are located on opposite sides of the pot [24]. Together, these findings suggest that belowground signals may contribute to the calibration of clasping behaviour and, potentially, to the plant’s response to support properties such as width and distance. In line with this interpretation, qualitative observations of root behaviour showed that roots consistently twisted around the buried portion of the support in all experimental conditions (Fig. 8A-E), as previously reported [24], suggesting a close interaction between the root system and the support structure. Although the present study was not designed to directly test the underlying physiological mechanisms, these observations support the possibility that belowground signals contribute to the regulation of clasping movements and the adjustment of their kinematics to support characteristics.

Regardless of the precise physiological mechanisms involved, the behavioural outcomes have demonstrated that movement duration varies systematically as a function of support characteristics. This finding suggests that climbing movements are not executed according to a fixed developmental program but are flexibly adjusted to the properties of the environment. The extent to which these adjustments reflect adaptive processes rather than growth-related constraints remains difficult to disentangle from the present data.

## Future directions, limitations and conclusion

The present study shows that pea plants flexibly adjust MT in response to task difficulty. However, the structure of this relationship differs from the classical formulation of Fitts’s law, requiring an organism-specific reinterpretation. Future research should evaluate whether the proposed descriptive index provides advantages over simpler formulations based directly on distance and support width, and whether its explanatory value generalizes across a wider range of task conditions, species, and environmental contexts to assess its generality. Expanding the spatial parameters would also allow incorporation of biologically meaningful constraints, such as maximum reachable distance, to refine the model and further clarify how task difficulty shapes plant movement. At the mechanistic level, additional work is needed to clarify how spatial cues are transmitted and integrated across root and shoot systems. Combining kinematic analyses with physiological measures of signalling dynamics may help uncover the distributed control processes underlying plant movement. Several limitations should be acknowledged. First, observations were terminated when the tendril contacted the support rather than at a fixed developmental age. Although this endpoint was chosen because it corresponds to the completion of the approach-to-clasp phase, variation in plant age at the time of contact may have introduced additional variability among individuals. Second, unlike classical Fitts’s law paradigms, the present study did not include an explicit measure of movement accuracy and speed. Consequently, it cannot be determined whether plants simultaneously regulate movement speed and accuracy, nor whether the observed adjustments reflect optimization processes. Future studies should therefore incorporate measures of clasping precision and contact success to evaluate whether plant movements exhibit speed-accuracy trade-off. Third, only two width and distance levels were included in the present experiment, which restricts the ability to characterize the functional relationship between task difficulty and MT. Future studies should therefore examine a wider range of support dimensions to determine whether the observed effect is maintained across different contexts. Fourth, because the analyses were restricted to successful approach-to-clasp behavior, the results cannot be generalized to the probability of successful support approaching, nor do they allow assessment of whether the experimental conditions differentially affected success rates. Future studies should determine whether the same spatial constraints that influence MT also affect the likelihood of successfully approaching and clasping a support. In conclusion, pea plants modulated MT according to spatial constraints. These adjustments cannot be fully explained only by differences in growth-related traits, suggesting that movement duration reflects more than the simple growth required to approach a target. While the mechanisms underlying this modulation remain to be clarified, the present findings suggest that pea plant movements are responsive to their environment and provide a basis for future investigations into the principles governing the organization of plant behaviour.

## Supplementary Information

Below is the link to the electronic supplementary material.

Supplementary Material 1

## Data Availability

All data presented in the study are openly available in Zenodo at: https://doi.org/10.5281/zenodo.18784260.
