# Supplementary material for: Temporal dynamics of plant movement under task constraints
Source: Mov Ecol. 2026 Jul 10;14:67. doi: 10.1186/s40462-026-00680-8 (PMC13647748; doi:10.1186/s40462-026-00680-8)
Supplement: Supplementary file 1 [file 40462_2026_680_MOESM1_ESM.docx]

**Supplementary materials**

**Temporal Dynamics of Plant Movement Under Task Constraints**

Silvia Guerra^1^*, Valentina Simonetti^1^, Emanuele Cannizzo^1^ & Umberto Castiello^1^

^1^Department of General Psychology, University of Padova, Padova, Italy

* Corresponding author: Silvia Guerra; Department of General Psychology, University of Padova, Padova, Italy; [silvia.guerra@unipd.it](mailto:silvia.guerra@unipd.it)

The file contains:

- Supplementary Tables S1 - S22

**Table S1.** Mean (± SD) of the plant age, height, average stem elongation, the length of the primary root, length of, and the number of the tendrils and for each experimental condition (Sn, Sf, Ln, Lf).

| **Experimental conditions** | Sf | Sn | Lf | Ln |
| --- | --- | --- | --- | --- |
| N° of individuals | 10 | 10 | 10 | 10 |
| Age (days) | 15.6 (2.32) | 10.3 (1.57) | 15.6 (4.43) | 11.2 (2.10) |
| Height (cm) | 22 (6.39) | 16.10 (3.04) | 29.63 (14.93) | 18.98 (6.88) |
| Average Stem elongation rate (cm/day) | 1.40 (.33) | 1.58 (.28) | 1.82 (.57) | 1.66 (.32) |
| Length of the primary root (cm) | 25.47 (6.51) | 21.76 (7.05) | 27.28 (6.22) | 19.79 (3.42) |
| N° of tendrils | 2.65 (.71) | 1.3 (.49) | 2.52 (.81) | 2 (.89) |
| Length of the tendrils (cm) | 5.29 (1.3) | 7.15 (.75) | 6.21 (1.03) | 7.18 (.99) |

***Note****.* Values are reported as mean with standard deviation given in parentheses. The average stem elongation rate was calculated as final stem height divided by the duration of the experimental session (days). Plant age and average stem elongation rate are reported for descriptive purposes only and were not included in the statistical analyses. Lf= Large-far condition; Ln= Large-near condition; Sf = Small-far condition; Sn =Small-near condition.

*Testing MT and spatial constraints in plants: A Fitts law inspired perspective*

**Table S2.** Mean (± SD) movement time (MT) for each experimental condition (Sn, Sf, Ln, Lf) and corresponding Index of Difficulty (ID) computed according to the classical formulation of Fitts’ law.

| **Experimental conditions** | **Movement time (min)** | **Index of Difficulty (ID)** |
| --- | --- | --- |
| Small-Far condition (Sf) | 3074.22 (838.50) | 4.18 |
| Small-Near condition (Sn) | 2268.5 (405.89) | 3.18 |
| Large-Far scondition (Lf) | 4034.43 (1115.22) | 3.32 |
| Large-Near condition (Ln) | 2610.56 (546.85) | 2.32 |

**Table S3.** Linear mixed-effects models testing the effects of distance (*A*) and target width (*W*) on movement time (MT), and their interaction, to assess deviations from the classical formulation of Fitts’ law. MT was the dependent variable. *A* and *W* were included as fixed effects, and Box was included as a random intercept to account for shared environmental variance.

|  | **β** | **SE** | **t** | **df** | **p** | **Marginal R2** | **Conditional R2** |
| --- | --- | --- | --- | --- | --- | --- | --- |
| ***MT ~ Log_2_(A) + Log_2_(W)***  Intercept | -661.1 | 761.1 | -.87 | 40 | .390 |  |  |
| Log_2(_A)  Log_2_(W) | 1179.0  774.6 | 258.8  300.1 | 4.55  2.58 | 40  40 | <.001^***^  .014^*^ | 0.41 | 0.41 |
| ***MT ~ Log_2_(A)*Log_2_(W)***  Intercept | 561.5 | 1202.7 | .47 | 40 | .643 |  |  |
| Log_2(_A)  Log_2_(W) | 745.8  -1375.0 | 419.6  1685.0 | 1.78  -.82 | 40  40 | .083  .419 |  |  |
| Log_2(_A):Log_2_(W) | 761.7 | 587.9 | 1.30 | 40 | .202 | 0.44 | 0.44 |

**Note.** β = regression coefficient; SE= Standard Error; R2 = Coefficient of determination; df = Degrees of Freedom (Satterthwaite’s method). N observations = 40. ° = p <.100; * = p < .050; ** = p < .010; *** = p < .001. A= Distance from the target; W=Width of the target. MT = Movement time.

**Table S4.** Model comparison

| **Model** | **Fixed effect** | **npar** | **AIC** | **ΔAIC** |
| --- | --- | --- | --- | --- |
| Fitts | ID | 4 | 677.03 | 16.9 |
| Additive | Log_2_(A) + Log_2_(W) | 5 | 660.10 | 0 |
| Interaction | Log_2_(A) * Log_2_(W) | 6 | 660.46 | 0.36 |

**Note.** ID=Index of difficulty; A = Distance from the target; W = Width of the target; npar= number of estimated parameters; AIC = Akaike’s Information Criterion; ΔAIC = difference in AIC relative to the best-fitting model.

*Testing whether MT modulation in pea plants relates to a biological cost of growth or an exploratory control strategy.*

**Table S5.** Linear mixed-effects model was fitted to test the effects of Fitts’ Index of Difficulty, plant height, and the length of the primary root length (PR) on movement time (MT). MT was included as the dependent variable. ID, Height, and PR were considered as fixed effects, and Box was included as a random intercept to account for shared environmental variance.

|  | **β** | **SE** | **t** | **df** | **p** | **Marginal R2** | **Conditional R2** |
| --- | --- | --- | --- | --- | --- | --- | --- |
| ***MT ~***  Intercept | 482.74 | 847.14 | .57 | 36.98 | .572 |  |  |
| ID  Height  PR | 248.06  44.84  37.93 | 246.21  14.74  24.54 | 1.01  3.04  1.55 | 34.54  36.27  33.40 | .321  .004^**^  .132 | 0.29 | 0.31 |

**Note.** β = regression coefficient; SE= Standard Error; R2 = Coefficient of determination; df = Degrees of Freedom (Satterthwaite’s method). N observations = 38. ° = p <.100; * = p < .050; ** = p < .010; *** = p < .001. A= Distance from the target; W=Width of the target. MT = Movement time; PR= length of the primary root.

**Table S6.** Linear mixed-effects models testing the effects of both the morphological traits across experimental conditions (Lf, Ln, Sf, Sn). Conditions were included as fixed effects, and Box was included as a random intercept to account for shared environmental variance. Height and the length of the primary root (PR) were the dependent variables.

|  | **β** | **SE** | **t** | **df** | **p** | **Marginal R2** | **Conditional R2** |
| --- | --- | --- | --- | --- | --- | --- | --- |
| ***Height ~***  Intercept  Condition_Ln | 29.63  -10.65 | 2.68  3.80 | 11.04  -2.80 | 40  40 | <.001^***^  .008^**^ |  |  |
| Condition_Sf  Condition_Sn | -7.63  -13.54 | 3.80  3.80 | -2.01  -3.57 | 40  40 | .051  <.001^***^ | .27 | .27 |
| ***PR ~***  Intercept  Condition_Ln | 27.28  -7.49 | 1.78  2.51 | 15.36  -2.98 | 38  38 | <.001^***^  .005^**^ |  |  |
| Condition_Sf  Condition_Sn | -1.80  -5.52 | 2.66  2.51 | -.68  -2.20 | 38  38 | .050  .034^*^ | .23 | .23 |

**Note.** β = regression coefficient; SE= Standard Error; R2 = Coefficient of determination; df = Degrees of Freedom (Satterthwaite’s method). Height: N observations = 40. PR: N observations = 38 ° = p <.100; * = p < .050; ** = p < .010; *** = p < .001. Ln= Large-near condition; Sf= Small-far condition; Sn= Small-near condition; PR= length of the primary root.

**Table S7.** Post-hoc pairwise comparisons for the effect of the Height and the length of the primary root (PR) across experimental conditions.

|  | **estimate** | **SE** | **df** | **t ratio** | **p** |
| --- | --- | --- | --- | --- | --- |
| ***Height ~***  Lf -Ln  Lf -Sf | 10.65  7.63 | 4.26  4.41 | 44.3  37.5 | 2.50  1.73 | .073  .322 |
| Lf - Sn  Ln -Sf  Ln-Sn  Sf -Sn | 13.54  -3.02  2.89  5.90 | 4.22  4.15  4.10  4.16 | 44.3  43.4  40.4  43.4 | 3.21  -.73  .70  1.42 | .013^*^  .886  .895  .495 |
| ***PR ~***  Lf -Ln  Lf -Sf | 7.49  1.80 | 2.82  3.10 | 42.3  36.6 | 2.65  .58 | .052  .937 |
| Lf - Sn  Ln -Sf  Ln-Sn  Sf -Sn | 5.52  -5.68  -1.97  3.71 | 2.80  2.92  2.72  2.91 | 42.3  41.0  38.4  39.9 | 1.97  -1.95  -.72  1.30 | .214  .224  .887  .582 |

**Note.** SE= Standard Error; df = Degrees of Freedom (Satterthwaite’s method). Height: N observations = 40. PR: N observations = 38; ° = p <.100; * = p < .050; ** = p < .010; *** = p < .001. Ln= Large-near condition; Sf= Small-far condition; Sn= Small-near condition; PR= Length of the primary root.

**Table S8.** Linear mixed-effects models testing the effects of both the height and the length of primary root (PR) on movement time (MT) across experimental conditions (Lf, Ln, Sf, Sn). Conditions were included as fixed effects, and Box was included as a random intercept to account for shared environmental variance. MT was the dependent variable and the height and vigor were included as covariates.

|  | **β** | **SE** | **t** | **df** | **p** | **Marginal R2** | **Conditional R2** |
| --- | --- | --- | --- | --- | --- | --- | --- |
| ***MT ~***  Intercept  Condition_Ln | 3625.37  -1311.23 | 920.69  447.08 | 3.94  -2.93 | 38  38 | <.001^***^  .006^**^ |  |  |
| Condition_Sf  Condition_Sn  Height  PR | -692.11  -1611.79  15.94  3.54 | 403.16  448.33  15.40  23.76 | -1.72  -3.59  1.03  .149 | 38  38  38  38 | .094  <.001^***^  .307  .882 | .47 | .47 |

**Note.** β = regression coefficient; SE= Standard Error; R2 = Coefficient of determination; df = Degrees of Freedom (Satterthwaite’s method). N observations = 38. ° = p <.100; * = p < .050; ** = p < .010; *** = p < .001. Ln= Large-near condition; Sf= Small-far condition; Sn= Small-near condition; MT = Movement time; PR=length of the primary root.

**Table S9.** Post-hoc pairwise comparisons for the effect of the height and the length of the primary root across experimental condition on movement time (MT).

|  | **estimate** | **SE** | **df** | **t ratio** | **p** |
| --- | --- | --- | --- | --- | --- |
| ***MT ~ Condition + Height + PR***  Lf -Ln  Lf -Sf | 1311  692 | 513  482 | 45.1  39.8 | 2.55  1.44 | .065  .484 |
| Lf - Sn  Ln -Sf  Ln-Sn  Sf -Sn | 1612  -619  301  920 | 513  449  400  447 | 45.1  40.1  40.0  40.8 | 3.14  -1.38  .75  2.06 | .015^*^  .520  .876  .184 |

**Note.** SE= Standard Error; df = Degrees of Freedom (Satterthwaite’s method). N observations = 38. ° = p <.100; * = p < .050; ** = p < .010; *** = p < .001. Ln= Large-near condition; Sf= Small-far condition; Sn= Small-near condition; MT = Movement time. PR= length of the primary root.

**Table S10.** Linear mixed-effects model was fitted to test the effects of Fitts’ Index of Difficulty, and tendril’s morphology (i.e., length and number of tendrils). MT was included as the dependent variable. ID, duration, length and number of tendrils were considered as fixed effects, and Box was included as a random intercept to account for shared environmental variance.

|  | **β** | **SE** | **t** | **df** | **p** | **Marginal R2** | **Conditional R2** |
| --- | --- | --- | --- | --- | --- | --- | --- |
| ***MT ~***  Intercept  ID  Length  N° of tendrils | 1530.06  414.56  4.39  -34.25 | 1801.87 297.46  15.91  196.37 | .85  1.39  .28  -.17 | 39.88  38.19  39.88  39.87 | .401  .171  .784  .862 | .05 | .07 |

**Note.** β = regression coefficient; SE= Standard Error; R2 = Coefficient of determination; df = Degrees of Freedom (Satterthwaite’s method). N observations = 40. ° = p <.100; * = p < .050; ** = p < .010; *** = p < .001. Ln= Large-near condition; Sf= Small-far condition; Sn= Small-near condition; MT = Movement time.

**Table S11.** Linear mixed-effects models testing the effects of the tendril’s morphology across experimental conditions (Lf, Ln, Sf, Sn). Conditions were included as fixed effects, and Box was included as a random intercept to account for shared environmental variance. The length and the number of the tendrils were considered as the dependent variables.

|  | **β** | **SE** | **t** | **df** | **p** | **Marginal R2** | **Conditional R2** |
| --- | --- | --- | --- | --- | --- | --- | --- |
| ***Lenght ~***  Intercept  Condition_Ln | 61.44  12.14 | 3.21  4.54 | 19.13  2.67 | 40  40 | <.001^***^  .011^*^ |  |  |
| Condition_Sf  Condition_Sn | -6.76  9.43 | 4.54  4.54 | -1.49  2.07 | 40  40 | .145  .044^*^ | .33 | .35 |
| ***N° tendrils~***  Intercept  Condition_Ln | .33  -.54 | .27  .39 | 1.2  -1.41 | 40  40 | .237  .165 |  |  |
| Condition_Sf  Condition_Sn | .23  -.99 | .39  .39 | .57  -2.55 | 40  40 | .575  .015^*^ | .23 | .23 |

**Note.** β = regression coefficient; SE= Standard Error; R2 = Coefficient of determination; df = Degrees of Freedom (Satterthwaite’s method). N observations = 40. ° = p <.100; * = p < .050; ** = p < .010; *** = p < .001. Ln= Large-near condition; Sf= Small-far condition; Sn= Small-near condition.

**Table S12.** Post-hoc pairwise comparisons for the effect of the length and the number of tendrils across experimental conditions.

|  | **estimate** | **SE** | **df** | **t ratio** | **p** |
| --- | --- | --- | --- | --- | --- |
| ***Length ~***  Lf -Ln  Lf -Sf | -12.15  6.76 | 5.06  5.27 | 44.4  42.1 | -2.40  1.28 | .091  .580 |
| Lf - Sn  Ln -Sf  Ln-Sn  Sf -Sn | -9.43  18.91  2.72  -16.19 | 5.04  4.92  4.84  4.93 | 44.5  41.9  38.3  41.8 | -1.87  3.84  .56  -3.28 | .254  .002^**^  .942  .011^*^ |
| ***N° of tendrils ~***  Lf -Ln  Lf -Sf | .56  -.22 | .43  .45 | 44.5  40.8 | 1.26  -.49 | .588  .962 |
| Lf - Sn  Ln -Sf  Ln-Sn  Sf -Sn | -99  -.75  .43  1.21 | .43  .42  .41  .42 | 44.5  42.9  38.9  42.3 | 2.29  -1.82  1.06  2.86 | .115  .277  .717  .032^*^ |

**Note.** SE= Standard Error; df = Degrees of Freedom (Satterthwaite’s method). N observations = 40. ° = p <.100; * = p < .050; ** = p < .010; *** = p < .001. Ln= Large-near condition; Sf= Small-far condition; Sn= Small-near condition; MT = Movement time.

**Table S13.** Linear mixed-effects model testing the effects of the tendril’s morphology (i.e., the length and the number of tendrils) on movement time (MT) across experimental conditions (Lf, Ln, Sf, Sn). Conditions were included as fixed effects, and Box was included as a random intercept to account for shared environmental variance. MT was the dependent variable, and the length and number of tendrils were included as covariates.

|  | **β** | **SE** | **t** | **df** | **p** | **Marginal R2** | **Conditional R2** |
| --- | --- | --- | --- | --- | --- | --- | --- |
| ***MT ~***  Intercept  Condition_Ln | 3706.85  -1877.85 | 850.44  360.64 | 4.36  -5.21 | 40  40 | <.001^***^  <.001^***^ |  |  |
| Condition_Sf  Condition_Sn  Length (mm)  Total nutations | -813.96  -2287.85  18.17  -299.37 | 337.98  365.15  11.57  148.61 | -2.41  -6.27  1.57  -2.01 | 40  40  40  40 | .021^*^  <.001^***^  .124  .051 | .53 | .53 |

**Note.** β = regression coefficient; SE= Standard Error; R2 = Coefficient of determination; df = Degrees of Freedom (Satterthwaite’s method). N observations = 40. ° = p <.100; * = p < .050; ** = p < .010; *** = p < .001. Ln= Large-near condition; Sf= Small-far condition; Sn= Small-near condition; MT = Movement time.

**Table S14.** Post-hoc pairwise comparisons for the effect of the number and length of tendrils on movement time (MT) across experimental conditions.

|  | **estimate** | **SE** | **df** | **t ratio** | **p** |
| --- | --- | --- | --- | --- | --- |
| ***MT ~***  Lf -Ln  Lf -Sf | 1878  814 | 424  400 | 44.7  43.4 | 4.43  2.03 | <.001^***^  .192 |
| Lf - Sn  Ln -Sf  Ln-Sn  Sf -Sn | 2288  -1064  410  1474 | 422  451  373  441 | 46.3  45.4  42.8  41.1 | 5.41  -2.36  1.10  3.34 | <.001^***^  .099  .693  .009^**^ |

**Note.** SE= Standard Error; df = Degrees of Freedom (Satterthwaite’s method). N observations = 40. ° = p <.100; * = p < .050; ** = p < .010; *** = p < .001. Ln= Large-near condition; Sf= Small-far condition; Sn= Small-near condition; MT = Movement time.

**Table S15.** Mean (± SD) of the duration (min), total and the length of circumnutation of plants for each experimental condition (Sn, Sf, Ln, Lf).

| **Experimental conditions** | **Duration of circumnutation (min)** | **Length of the circumnutation (mm)** | **N° of circumnutation** |
| --- | --- | --- | --- |
| Sf | 122.46 (19.98) | 55.34 (13.39) | 24.26 (8.20) |
| Sn | 93.49 (10.33) | 51.21 (11.44) | 23.33(5.99) |
| Lf | 131.07 (21.77) | 58.45 (11.31) | 32.05 (17.36) |
| Ln | 96.93 (17.73) | 46.40 (14.53) | 26.81 (8.32) |

***Note****.* Lf = Large-far condition; Ln= Large-near condition; Sf= Small-far condition; Sn= Small-near condition.

**Table S16.** Linear mixed-effects model was fitted to test the effects of Fitts’ Index of Difficulty, and circumnutation dynamics (i.e., duration, length and total of circumnutation). MT was included as the dependent variable. ID, duration, length and total of circumnutation were considered as fixed effects, and Box was included as a random intercept to account for shared environmental variance.

|  | **β** | **SE** | **t** | **df** | **p** | **Marginal R2** | **Conditional R2** |
| --- | --- | --- | --- | --- | --- | --- | --- |
| ***MT ~ Circumnutation dynamics***  Intercept  ID | 2510.04  216.18 | 837.88  221.84 | 3.00  .97 | 39.93  34.45 | .005^**^  .337 |  |  |
| Length  Duration  Total nutations | -4.18  .84  622.06 | 11.70  6.58  134.39 | -.36  .13  4.63 | 39.62  35.78  36.82 | .723  .898  <.001^***^ | .38 | .46 |

**Note.** β = regression coefficient; SE= Standard Error; R2 = Coefficient of determination; df = Degrees of Freedom (Satterthwaite’s method). N observations = 40. ° = p <.100; * = p < .050; ** = p < .010; *** = p < .001. Ln= Large-near condition; Sf= Small-far condition; Sn= Small-near condition; MT = Movement time.

**Table S17.** Linear mixed-effects models testing the effects of the circumnutation dynamics across experimental conditions (Lf, Ln, Sf, Sn). Conditions were included as fixed effects, and Box was included as a random intercept to account for shared environmental variance. Duration, length and the number of the circumnutations were considered as the dependent variables.

|  | **β** | **SE** | **t** | **df** | **p** | **Marginal R2** | **Conditional R2** |
| --- | --- | --- | --- | --- | --- | --- | --- |
| ***Duration ~***  Intercept  Condition_Ln | 130.37  -37.27 | 5.73  7.32 | 22.77  -5.09 | 23.77  38.40 | <.001^***^  <.001^***^ |  |  |
| Condition_Sf  Condition_Sn | -11.60  -37.80 | 7.55  7.34 | -1.53  -5.15 | 39.99  38.86 | .13  <.001^***^ | .47 | .52 |
| ***Lenght ~***  Intercept  Condition_Ln | 55.20  -7.36 | 4.05  5.15 | 13.62  -1.43 | 20.95  34.60 | <.001^***^  .161 |  |  |
| Condition_Sf  Condition_Sn | .15  -3.98 | 5.31  5.16 | .03  -.77 | 36  35 | .977  .446 | .06 | .20 |
| ***Total nutations ~***  Intercept  Condition_Ln | .62  -.80 | .29  .41 | 2.27  -2.09 | 40  40 | .028^*^  .043^*^ |  |  |
| Condition_Sf  Condition_Sn | -.38  -1.30 | .41  .41 | -.98  -3.37 | 40  40 | .330  .001^**^ | .24 | .24 |

**Note.** β = regression coefficient; SE= Standard Error; R2 = Coefficient of determination; df = Degrees of Freedom (Satterthwaite’s method). N observations = 40. ° = p <.100; * = p < .050; ** = p < .010; *** = p < .001. Ln= Large-near condition; Sf= Small-far condition; Sn= Small-near condition.

**Table S18.** Post-hoc pairwise comparisons for the effect of the Duration and the number of circumnutation across experimental conditions.

|  | **estimate** | **SE** | **df** | **t ratio** | **p** |
| --- | --- | --- | --- | --- | --- |
| ***Duration ~***  Lf -Ln  Lf -Sf | 37.27  11.60 | 7.98  8.43 | 42.2  44.5 | 4.67  1.38 | <.001^***^  .521 |
| Lf - Sn  Ln -Sf  Ln-Sn  Sf -Sn | 37.80  -25.67  .57  26.20 | 8.02  7.72  7.49  7.71 | 42.8  39.8  36.8  39.6 | 4.72  -3.32  .07  3.40 | <.001^***^  .010^*^  .999  .008^**^ |
| ***Total nutations ~***  Lf -Ln  Lf -Sf | .80  .38 | .43  .45 | 44.3  37.5 | 1.86  .85 | .258  .831 |
| Lf - Sn  Ln -Sf  Ln-Sn  Sf -Sn | 1.30  -.43  -.49  .92 | .43  .42  .42  .42 | 44.3  43.4  40.4  43.4 | 3.03  -1.01  1.18  2.17 | .020^*^  .744  .642  .147 |

**Note.** SE= Standard Error; df = Degrees of Freedom (Satterthwaite’s method). N observations = 40. ° = p <.100; * = p < .050; ** = p < .010; *** = p < .001. Ln= Large-near condition; Sf= Small-far condition; Sn= Small-near condition.

**Table S19.** Linear mixed-effects model testing the effects of the circumnutation dynamics (i.e., duration, length and total of circumnutation) on movement time (MT) across experimental conditions (Lf, Ln, Sf, Sn). Conditions were included as fixed effects, and Box was included as a random intercept to account for shared environmental variance. MT was the dependent variable and length, duration and total nutation were included as covariates.

|  | **β** | **SE** | **t** | **df** | **p** | **Marginal R2** | **Conditional R2** |
| --- | --- | --- | --- | --- | --- | --- | --- |
| ***MT ~***  Intercept  Condition_Ln | 6915.33  -2015.18 | 922.97  393.04 | 7.49  -5.13 | 39.99  39.99 | <.001^***^  <.001^***^ |  |  |
| Condition_Sf  Condition_Sn  Length (mm)  Duration (min)  Total nutations | -1020.25  -2238.52  -4.46  -20.83  325.56 | 305.36419.00  8.90  6.42  116.80 | -3.34  -5.34  -.50  -3.25  2.79 | 37.55  39.76  39.87  39.70  35.15 | .002^**^  <.001^***^  .619  .002^**^  .008^**^ | .65 | .70 |

**Note.** β = regression coefficient; SE= Standard Error; R2 = Coefficient of determination; df = Degrees of Freedom (Satterthwaite’s method). N observations = 40. ° = p <.100; * = p < .050; ** = p < .010; *** = p < .001. Ln= Large-near condition; Sf= Small-far condition; Sn= Small-near condition; MT = Movement time.

**Table S20.** Post-hoc pairwise comparisons for the effect of the duration, length and total nutation on MT across experimental condition.

|  | **estimate** | **SE** | **df** | **t ratio** | **p** |
| --- | --- | --- | --- | --- | --- |
| ***MT ~***  Lf -Ln  Lf -Sf | 2015  1020 | 458  363 | 48.5  46.7 | 4.40  2.81 | <.001^***^  .035^*^ |
| Lf - Sn  Ln -Sf  Ln-Sn  Sf -Sn | 2239  -995  223  1218 | 491  367  308  388 | 48.5  40.0  39.8  40.7 | 4.56  -2.71  .73  3.14 | <.001^***^  .046^*^  .886  .016^*^ |

**Note.** SE= Standard Error; df = Degrees of Freedom (Satterthwaite’s method). N observations = 40. ° = p <.100; * = p < .050; ** = p < .010; *** = p < .001. Ln= Large-near condition; Sf= Small-far condition; Sn= Small-near condition; MT = Movement time.

*Modeling the Effect of Spatial Constraints in Pea Plant Movements.*

**Table S21.** Linear mixed-effects models testing the effect of spatial constraints in pea plant movements. Movement time (MT) was the dependent variable, ID was included as a fixed effect, and Box was included as a random intercept to account for shared environmental variance.

|  | **β** | **SE** | **t** | **df** | **p** | **Marginal R2** | **Conditional R2** |
| --- | --- | --- | --- | --- | --- | --- | --- |
| **MT *~ ID_Fitts_*** |  |  |  |  |  |  |  |
| Intercept IDfitts | 1934.82  358.59 | 822.20  246.84 | 2.35  1.45 | 39.66  38.22 | .024^*^  .154 | 0.05 | 0.08 |
| **MT *~ ID_plant_*** |  |  |  |  |  |  |  |
| Intercept ID_plant_ | 1032.3 2150.1 | 415.2 409.1 | 2.49  5.27 | 40  40 | .017^*^  <.001^***^ | 0.41 | 0.41 |

**Note.** β = regression coefficient; SE= Standard Error; R2 = Coefficient of determination; df = Degrees of Freedom (Satterthwaite’s method). N observations = 40. ° = p <.100; * = p < .050; ** = p < .010; *** = p < .001.

**Table S22.** Model comparison

| **Model** | **Fixed effect** | **npar** | **AIC** | **ΔAIC** |
| --- | --- | --- | --- | --- |
| ID_Fitts_ | ID= log₂(2A/W) | 4 | 677.03 | 19.05 |
| ID_plant_ | ID = log₂(1 + (A*W)/k) | 4 | 657.98 | 0 |

**Note.** ID=Index of difficulty; A = Distance from the target; W = Width of the target; k=the product between the averages of target distance and width across experimental conditions; npar= number of estimated parameters; AIC = Akaike’s Information Criterion; ΔAIC = difference in AIC relative to the best-fitting model.
